# The NULevel trial of a scalable, technology-assisted weight loss maintenance intervention for obese adults after clinically significant weight loss: study protocol for a randomised controlled trial

**DOI:** 10.1186/s13063-015-0931-7

**Published:** 2015-09-22

**Authors:** Elizabeth H. Evans, Vera Araújo-Soares, Ashley Adamson, Alan M. Batterham, Heather Brown, Miglena Campbell, Stephan U. Dombrowski, Alison Guest, Daniel Jackson, Dominika Kwasnicka, Karim Ladha, Elaine McColl, Patrick Olivier, Alexander J. Rothman, Kirby Sainsbury, Alison J. Steel, Ian Nicholas Steen, Luke Vale, Martin White, Peter Wright, Falko F. Sniehotta

**Affiliations:** Institute of Health & Society, Newcastle University, Baddiley-Clark Building, Richardson Road, Newcastle upon Tyne, NE2 4AX UK; Fuse, the UK CRC Centre for Translational Research in Public Health, Institute of Health & Society, Newcastle University, Baddiley-Clark Building, Richardson Road, Newcastle upon Tyne, NE2 4AX UK; Human Nutrition Research Centre, Institute of Health & Society, University of Newcastle, William Leech Building, Medical School, Framlington Place, Newcastle upon Tyne, NE2 4HH UK; Health and Social Care Institute, Teesside University, Parkside West, Middlesbrough Tees Valley, TS1 3BA UK; School of Natural Sciences, Division of Psychology, University of Stirling, Stirling, FK9 4LA UK; Open Lab, School of Computing Science, Newcastle University, 89 Sandyford Road, Newcastle upon Tyne, NE1 8HW UK; Department of Psychology, University of Minnesota, 75 East River Road, Minneapolis, MN 55455 USA; Centre for Diet and Activity Research (CEDAR), MRC Epidemiology Unit, School of Clinical Medicine, University of Cambridge, Institute of Metabolic Science, Cambridge Biomedical Campus, Cambridge, CB2 0QQ UK; Newcastle Clinical Trials Unit, Newcastle University, 1-4 Claremont Terrace, Newcastle upon Tyne, NE2 4AE UK

**Keywords:** Behaviour, Randomised controlled trial, Clinical protocol, Obesity, Overweight, Weight loss, Weight loss maintenance

## Abstract

**Background:**

Effective weight loss interventions are widely available but, after weight loss, most individuals regain weight. This article describes the protocol for the NULevel trial evaluating the effectiveness and cost-effectiveness of a systematically developed, inexpensive, scalable, technology-assisted, behavioural intervention for weight loss maintenance (WLM) in obese adults after initial weight loss.

**Methods/Design:**

A 12-month single-centre, two-armed parallel group, participant randomised controlled superiority trial is underway, recruiting a total of 288 previously obese adults after weight loss of ≥5 % within the previous 12 months. Participants are randomly assigned to intervention or control arms, with a 1:1 allocation, stratified by sex and percentage of body weight lost (<10 % vs ≥10 %). Change in weight (kg) from baseline to 12 months is the primary outcome. Weight, other anthropometric variables and 7-day physical activity (assessed via accelerometer) measures are taken at 0 and 12 months. Questionnaires at 0, 6 and 12 months assess psychological process variables, health service use and participant costs. Participants in the intervention arm initially attend an individual face-to-face WLM consultation with an intervention facilitator and then use a mobile internet platform to self-monitor and report their diet, daily activity (via pedometer) and weight through daily weighing on wirelessly connected scales. Automated feedback via mobile phone, tailored to participants’ weight regain and goal progress is provided. Participants in the control arm receive quarterly newsletters (via links embedded in text messages) and wirelessly connected scales. Qualitative process evaluation interviews are conducted with a subsample of up to 40 randomly chosen participants. Acceptability and feasibility of procedures, cost-effectiveness, and relationships among socioeconomic variables and WLM will also be assessed.

**Discussion:**

It is hypothesised that participants allocated to the intervention arm will show significantly lower levels of weight regain from baseline than those in the control arm. To date, this is the first WLM trial using remote real-time weight monitoring and mobile internet platforms to deliver a flexible, efficient and scalable intervention, tailored to the individual. This trial addresses a key research need and has the potential to make a vital contribution to the evidence base to inform future WLM policy and provision.

**Trial registration:**

http://www.isrctn.com/ISRCTN14657176 (registration date 20 March 2014).

## Background

Obesity remains a major public health concern worldwide [1]. It is strongly associated with chronic illness, sickness, absence from work, reduced life expectancy and costs the UK economy £16 billion per year [2]. The prevalence of obesity and consequent ill health are more marked amongst the most socioeconomically deprived, contributing to health inequalities [1]. It is commonly recommended that obese adults aim for a clinically significant weight loss of at least 5–10 % body weight [3].

Behavioural weight loss interventions typically produce initial, clinically significant weight loss [4, 5] and such evidence-based interventions are now widely available [6]. However, most people regain a third of the lost weight within a year and the rest within 3–5 years [4]. The health benefits of weight loss can only be achieved if it is maintained over the longer term [7]. Helping people to avoid weight regain after successful weight loss is therefore vital to tackle obesity and its consequences. Although evidence suggests that extended care interventions can slow down weight regain, there is to date a lack of scalable evidence-based interventions to support people after successful weight loss [8, 9]. It is currently not known how best to support individuals who have lost weight to prevent or slow down weight regain [10]. This gap in the evidence limits the impact of services that elicit the initial weight loss.

### Rationale and development of the NULevel intervention

The NULevel intervention was developed, following Medical Research Council (MRC) guidance for the development of complex interventions [11], to provide scalable weight loss maintenance (WLM) support (i.e. an intervention that is inexpensive, independent of location and specific resources, flexible and tailored to individual support needs). To achieve this aim, NULevel utilises an automated remote weight-monitoring and feedback system using participants’ mobile phones as the main mode of delivery. Standard digital body weight scales have been modified to transmit body weight data to a central server through the mobile phone network in real time. The bespoke NULevel interface stores and processes incoming information to provide both automated feedback for the participant and a data hub for the intervention team. The use of personal weighing scales is central to NULevel. Frequent self-weighing is associated with better WLM [12–14]. In NULevel, regular self-weighing enables personal tailoring of intervention content based on an underlying ‘traffic light’ system, allowing the intervention to be less intensive when the participant’s body weight is stable and more intensive at times when body weight is increasing [15]. Mobile phones were chosen as a flexible and ubiquitous digital communication platform (e.g. 93 % of adults in the UK personally own/use a mobile phone [16]), which allow access to text messages and mobile internet content, as well as enabling communication with the intervention delivery team. In NULevel, mobile phones are also used to tailor the content and intensity of the intervention in response to participant-provided information. Participants are prompted once a week to report their step counts, progress on personalized eating goals, mood, goal priority, satisfaction with and confidence for weight loss maintenance. They can also request further contact with the intervention team.

NULevel was developed based on a recent systematic review of randomised controlled trials (RCTs) of WLM interventions for obese adults after clinically significant weight loss [9]. The review found that complex interventions, targeting both dietary and physical activity behaviours, significantly reduced weight regain. There was no evidence for a dose–response relationship. Moreover, no evidence for the effectiveness of exclusively internet-delivered interventions was found [9]. Formative qualitative research for this project suggested that an initial face-to-face contact with the intervention provider may increase participants’ motivation to engage with internet-delivered elements of a complex intervention [17]. NULevel starts, therefore, with an individual face-to-face WLM consultation to facilitate the transition from weight loss to WLM.

The NULevel content was informed by further analyses of the review data to identify theories, behaviour change techniques and intervention components associated with effective interventions [9], a systematic review of theories of behavioural maintenance [18], evidence from the US National Weight Control Registry [19], qualitative research [17, 20, 21] and intensive user-centred design.

### Current trial

The NULevel trial is the first RCT of a systematically developed WLM intervention utilising remote real-time weight monitoring and mobile internet platforms to deliver a scalable intervention, tailored to the individual. With a few exceptions (e.g. [15, 22, 23]), most previous WLM trials induced weight loss before randomisation. NULevel is recruiting initially obese individuals who have lost weight through a range of different strategies, including self-directed efforts and commercial weight loss programmes.

The primary aim of NULevel is to evaluate the effectiveness of an inexpensive, scalable, technology-assisted, behavioural intervention for reducing weight regain among obese adults after initial weight loss. Secondary objectives are: (1) to test the acceptability and feasibility of the trial procedures through an integrated internal pilot study involving the first 50 participants in the trial, (2) to estimate the effectiveness and cost-effectiveness of the behavioural intervention in maintaining weight loss, and (3) to elucidate any associations among socioeconomic variables and WLM.

## Methods/Design

**Fig. 1:**
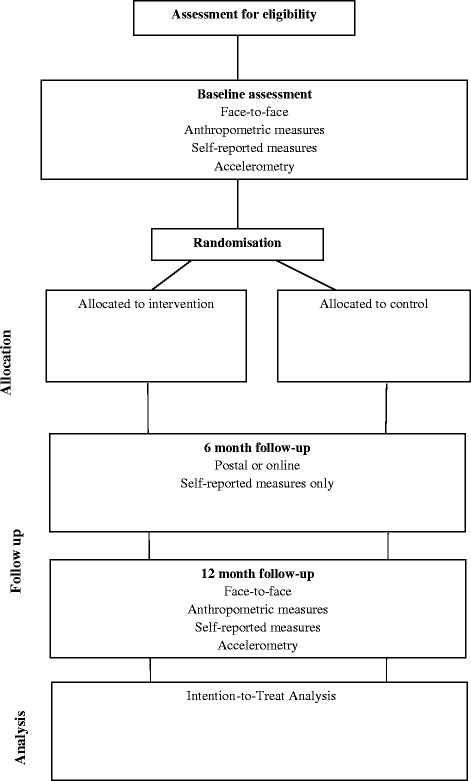
Planned recruitment, allocation and follow-up for the NULevel trial

This is a single-centre, two-armed, parallel group, participant randomised controlled superiority trial designed to compare the effectiveness and cost-effectiveness of a newly developed WLM intervention against a minimal intensity control condition over 12 months. Participant flow through the study is illustrated in Fig. 1. Ethics approval was obtained 6 February 2014 from the East Midlands-Derby National Research Ethics Service (REC: 14/EM/0069). The trial has been registered on the ISRCTN registry (ISRCTN14657176: http://www.controlled-trials.com/ISRCTN14657176).

### Participants and inclusion criteria

The aim is to recruit 288 adults (≥18 years). Individuals are eligible to participate if they meet the following eligibility criteria:

1. A body mass index (BMI) of ≥30 kg/m^2^ any time in the 24 calendar months preceding trial entry (i.e. the date of consent); the BMI threshold is ≥28 kg/m^2^ for individuals of South Asian descent [24];
2. A weight loss of ≥5 % in the 12 calendar months preceding trial entry. Written verification of this weight loss should be provided by a physician, weight loss counsellor or friend; if no such evidence from a third party is available, participants may self-certify their weight loss;
3. Ordinarily living or working in the North East of England;
4. Access to and willingness to use an internet-enabled mobile telephone in order to receive messages from the research team, containing embedded links to relevant online content;
5. Ability to use a standing scale for weight measurements.

Individuals are excluded from participation on the following grounds:

1. Participation in prior development studies of the intervention;
2. Weight loss due to illness or surgical procedures, including bariatric surgery;
3. Pregnancy or plans to become pregnant in the next year;
4. Breastfeeding an infant under 6 months of age;
5. Current involvement in other weight intervention research studies;
6. Inability to understand written material or telephone conversations in English;
7. A diagnosis of anorexia nervosa, bulimia nervosa or purging disorder, or of any condition which may preclude increasing mild to moderate physical activities such as walking;
8. Baseline weight of >175 kg (due to the measurement range of the provided scales);
9. Plans to leave the area or to undertake long-term travel in the forthcoming 12 months.

### Participant identification and recruitment

Participants are recruited via commercial weight loss providers (e.g. Slimming World and Lighter Life), local authority-commissioned adult weight management programmes, social media including Facebook and Twitter and other forms of community recruitment including web advertisements, and flyers and posters in local venues.

### Screening and consent

Interested individuals are asked to complete an online enquiry form, providing data to enable an initial eligibility assessment to be made. A research team member evaluates the provided information against the inclusion and exclusion criteria and informs the interested individual of their eligibility status via email. Individuals who provisionally meet the inclusion criteria then receive, by email, the study information sheet and a letter of invitation to attend a baseline appointment with a research team member, optionally at a university venue or at the participant’s home. At this appointment, their eligibility status is formally determined and they are offered the option of asking any questions about the study information sheet and/or their participation. Once any questions have been answered, the outcome assessor reiterates the conditions of consent, including that all data collected will remain confidential and they are free to withdraw at any time throughout the study. Participants then sign the consent form in the presence of the outcome assessor.

### Interventions

#### Allocation of wirelessly connected scales

At the end of the baseline assessment, every participant (intervention and control arm) receives a wirelessly connected scale. The scales have a standard digital display showing the user their current weight in their choice of units. For data transmission, the scales use the mobile phone network to avoid the need for any infrastructure or connectivity requirements in participants’ homes. To maximize coverage, a ‘multi-network’ SIM card allows the scales to select between multiple service providers based on the available signal. Even with multiple networks to choose from, mobile internet connectivity is not universal; instead, the basic GSM short message service (SMS) is used to transmit weights, which only requires that the scales can register with any network. Mobile phone technology requires significantly more power than standard scales, and battery-powered devices typically require regular recharging. The wirelessly connected scales were engineered to last for up to 12 months on a single set of batteries, and are therefore suitable for use.^1^

Every time participants weigh themselves, the recorded weight is transmitted over the mobile phone network to a central server and delivered into the software used to administer the digital elements of the intervention in real time. Weights are automatically recorded, dated and time-stamped for each participant. Participants are informed that only those randomised to the intervention arm receive feedback on their weight progress.

### The NULevel intervention

The intervention is delivered using a combination of a single face-to-face contact; automated messages in response to weighing data and weekly questionnaires delivered as mobile web content (text message with internet link) and text messages generated by the intervention team. On request of the participant, individual telephone calls with a member of the research team can be scheduled to discuss specific problems with WLM. NULevel is primarily based on self-regulation theory [25]. It uses technology to facilitate the monitoring of weight, behavioural goals and risk factors for lapses and it provides feedback and reinforcement. As such it is based on effective behavioural principles [15, 26]. This approach also allows tailoring of intervention components to the participants’ progress.

#### Initial face-to-face WLM consultation

Each participant allocated to the intervention arm of the trial is invited to attend an initial individual face-to-face WLM consultation with a psychologist, trained to deliver the NULevel intervention (facilitator), either at the university site or another agreed venue. Prior to this session, each participant receives by post a weight management history questionnaire, an Omron III Walking Style pedometer (Omron UK Ltd, Milton Keynes, UK) and a food diary to record over 4 days (including at least one weekend day) what they eat and drink, and how many steps they take based on the pedometer readings. They bring the food diary, pedometer and completed questionnaire to their WLM consultation. The consultation takes between 60 and 90 minutes. The main aims of the session are to build rapport, to ensure that the participant has a clear, sustainable and healthy plan of how to keep the lost weight off, and to gather information from the participant to personalise subsequent intervention contacts. The detailed structure, objectives, and behaviour change techniques of the session are summarised in Table 1.

**Table 1:** Summary of the NULevel face-to-face weight loss maintenance consultation

Section	Content/objectives	Behaviour change techniques
Introduction (5 mins)	Welcome and consent; explain session structure	
A: Weight history and goals (10 mins)	Review weight history, overall trajectory and number of WL attempts	Prompting focus on past success
	Agree overall weight goal and regain thresholds for red and yellow zones	Goal setting (outcome)
	Demonstrate and encourage weight self-monitoring using online study interface; explain weight-related SMS feedback system	Prompt self-monitoring of behavioural outcome
B: Weight strategies and success (10 mins)	Review current weight management strategies and future preferences	Selection of behavioural options
	Identify individual WL success factors: initial trigger(s); motivations; duration; regain/setbacks; consequences	Prompt focus on past success
C: Dietary review (10 mins)	Review eating behaviours using 4-day food diary (habits,frequency; nutritional adequacy; context; any uncontrolled eating; whether participant desires any dietary changes)	Provide feedback on performance
		Goal setting (behaviour)
	Identify specific foods/drinks that participant overeats and finds hard to control/avoid (trigger foods); identify less problematic alternatives	Barrier identification
		Problem solving
D: Set eating goals (10 mins)	Identify and formulate ≥1 SMART eating goals for WLM, specifying how, where and when behaviour is performed, potential sources of social support, barriers, and possible solutions	Goal setting (behaviour)
		Problem solving
	Agree plan against which participant monitors and evaluates own performance	Action planning
		Coping planning
		Plan social support/social change
	Discuss eating goal self-monitoring using online study interface Explain input of eating data to online ‘diary’, SMS feedback and goal review/resetting process	Prompt self-monitoring of behaviour
		Review of behavioural goals
E: Coping plans for tempting situations (5 mins)	Identify situations in which participant struggles to adhere to desired eating habits. Participant generates strategies to avoid this outcome	Barrier identification
		Problem solving
		Coping planning
	***Optional***: identify an alternative WLM plan, if participant desires, from three options: Mediterranean diet, calorie-controlled diet and Change4life	Provide instruction on how to perform the behaviour and information on where/when to perform the behaviour
	Discuss how, where, when and with whom to put the plan into place, possible obstacles to implementing the plan, and possible solutions to apply	Action planning
		Coping planning
		Barrier identification
		Problem solving
F: Physical activity review and goal setting (10 mins)	Review and discuss current physical activity	Provide feedback on performance
	Highlight importance of physical activity to WLM and overall health, and discuss ways to become more active, if desired	Provide information on the consequences of behaviour to the individual
	Identify and formulate a SMART physical activity goal for WLM (e.g. daily step count), specifying how, where and when behaviour is performed, potential sources of social support, barriers, and possible solutions	Goal setting (behaviour)
		Action planning
		Barrier identification
		Coping planning
		Plan social support/social change
	Discuss activity goal self-monitoring using online study interface Explain input of step counts to online ‘diary’, SMS feedback and goal review/resetting process	Prompt self-monitoring of behaviour
		Review of behaviour goals
G: Relapse prevention (10 mins)	Explain rationale for behavioural self-monitoring, lapse/relapse distinction and importance of problem solving	Prompt self-monitoring of behavioural determinant
	Explain weekly online diary contains questions on WLM confidence; health, wellbeing and priority placed on WLM in previous week Explain options to contact the research team (by email, SMS and diary tick-box)	Social support
	Identify situations in which participant struggles to be active Participant generates “if … then …” formulations using a volitional helpsheet (Armitage 2014) physically drawing a line to link situations with several possible solutions	Barrier identification
		Problem solving
		Coping planning
H: Summary	Summarise eating, physical activity and weight goals Check participant understanding of action plans, coping plans and self-monitoring procedures	
	Give participant written details of goals, online interface user guide, log-in details and (if desired) new WLM eating plan	
	Address remaining concerns, explain next steps and thank participant for their time	

*SMART* specific, measurable, attainable, relevant, time-based, *SMS* short message service, *WLM* weight loss maintenance

Participants discuss their weight history with the intervention facilitator. They are then supported to formulate and plan for specific weight, dietary and physical activity maintenance goals, and to undertake problem solving to identify likely barriers and possible solutions to achieving these maintenance goals. When setting weight goals, participants identify weight thresholds to define a personal ‘traffic light’ system to monitor their weight. The traffic light system helps to tailor the intervention to individual progress. They define a ‘green zone’, e.g. a desired weight corridor that indicates that their WLM is on target, a ‘yellow zone’ when weight is increasing and a ‘red zone’ when weight regain is substantial [15]. The thresholds for the yellow and red zone are usually set at 2.5 % and 5 % over the baseline weight respectively, but participants can personalise these thresholds in accordance to their body weight and previous weight loss. They can also readjust their traffic light system later if substantial weight changes occur. Participants learn that NULevel will offer more support to tackle regain and intervention contacts will become more frequent if their weight exceeds the yellow zone weight threshold for 4 consecutive days. Participants are encouraged to maintain existing healthy weight management practices if such practices are sustainable, and if the participant wishes. This may include the use of local authority or commercial weight loss services. When participants’ weight exceeds the red zone weight threshold for 4 consecutive days, a member of the intervention team initiates a review of current WLM goals and behaviours and provides additional support (see Table 2).

**Table 2:** Description of automated and manual SMS text messages in NULevel

Construct	Sent to	Frequency	Trigger	Automated/manual	Example
Theoretical themes of maintenance					
Motivation	All participants	Every 14 days	Specified number of days since joining study	Automated	Benefits of keeping weight off include better health, self-confidence, new clothes, more energy and a feeling of achievement. Think of what else you’d add to this list. Tell us what motivates you to maintain weight loss.
Resources					Sometimes we intend to be active but conclude ‘I don’t have time for this!’ Do you do this? If so, click: {link: web content}.
Self-regulation					Are you planning to eat out anytime soon? Thinking ahead helps you stay in control: what healthy option from the menu would you enjoy?
Habits					Creating new positive habits to keep weight off takes time; you have to repeatedly do things to build them into your routine (same place, same time, same way). Try to stay positive: habits take time to develop.
Environment and social influences					Eating with supportive family or friends is enjoyable and it can help keep your weight on track too. If you need some inspiration, here are some simple and delicious ideas: {link: web content}.
*Behaviour*	*Sent to*	*Frequency*	*Trigger*	*Automated/manual*	*Example*
Prompt self-monitoring of behavioural goals					
Physical activity and eating	All participants	Every 7 days	Specified weekday*	Automated	Please complete your diary today, by following this link: {link:diary}.
Provide feedback on behaviour					
Physical activity and eating	All participants	Every 7 days	Participant enters activity and eating goal data	Automated	Thanks for submitting your activity and eating goal diary entries - they help you keep on track to meet your weight goal.
					This week you missed your eating goals. Sticking to your plans is key to keeping the weight off! Follow this link {link:plan} to plan how, when and where you can meet your goals next week.
Self-weighing	All participants	Daily (days 1–7)	Participant self-weighs daily		Thanks for weighing yourself - it’s important for keeping the weight off!
		Weekly (day 8 →)			You weighed yourself regularly this week: well done and keep it up.
Prompt review of behavioural goals					
Physical activity and eating	All participants	As required	Participant meets behavioural goals on 3 consecutive wks	Manual	You have mastered your activity goals - congratulations! Why not set yourself some new goals for next week, since you’ve mastered these ones? If so, follow this link to write a new plan {link: edit plan}. If not, you need do nothing.
*Weight zone*	*Sent to*	*Frequency*	*Trigger*	*Automated/manual*	*Example*
Prompt self-monitoring of outcome (weight goal set in session)					
All	As required	As required	No weight in 96 hrs	Automated	Don’t forget to weigh yourself today - it’s important for maintaining your weight loss.
All	All	Every 6 weeks	Specific dates	Automated	Have you looked at your weight graph recently? If not, simply log in at {link: login} and click ‘weight’ to view your progress. Log in details not working or forgotten? Please text back and we’ll help!
Provide feedback on behavioural outcome					
Green	All participants	Every 7 days	Weight stable in green zone	Automated	This week you managed to stick to your green zone weight: good job.
Yellow/red	As required	As required	≥4 weights at or above yellow/red zone threshold	Manual	We noticed that you regained some of your lost weight and you are now in the yellow/red zone you specified at our support session. Could you please reply to this message to confirm that your last few weights are correct?
Problem solving, action planning and coping planning; prompt focus on past success					
Yellow/red	As required	2^nd^ day in yellow/red zone	Participant confirms accuracy of recent weights	Manual	Okay, thanks for letting us know your recent weights are correct. We’re here to help you get back on track. Would you like us to look at your diary entries and weight to produce some extra, positive, personalized feedback for you?
Yellow/red	As required	As required	≥4 weights below yellow/red zone threshold	Manual	You’re back in the green zone - congratulations for getting your weight back on track!
					You’re back in the yellow zone – congratulations!
Yellow/red	As required	2^nd^ day in yellow/red zone	Participant confirms accuracy of recent weights	Automated	What would you like to do about your regain? You may want to concentrate on just avoiding further regain, or instead to focus on losing the weight you’ve gained. We’re here to support you, whatever you prefer. Please text us your preference.
Red	As required	3^rd^ day in red zone	Participant confirms wish to lose weight	Automated	You’ve lost weight before, so you know a lot about which methods work for you and which don’t. With that in mind, do you currently have a plan for how you’d like to lose weight? Alternatively, would you like our help in developing one? Please text back to let us know.
Red	As required	4^th^ day in red zone; unscripted exchanges continue thereafter until weight loss achieved	Participant requests ongoing support in weight loss	Automated	It’s great to hear that you’d like our help in developing a plan to get your weight back on track. You’re an expert on strategies that worked for you before - would you like your new plan to involve these?
					We’d like to ask you more about your eating and activity over the last few weeks. This will help pinpoint the causes of your regain.
					What would you say has affected your weight the most, recently?
Social support					
Yellow/red	As required	As required	Participant requests ongoing support in weight loss	Manual	We are glad to hear that you’d like our help in developing a plan to get your weight back on track. This is done most easily with a phone call: please text back CALL ME if you’d like a call, with a suitable day and time. Please text NO CALL if you don’t want us to ring: we’ll continue to support your plan development by SMS.
Prompt review of outcome goal(s)					
Green	As required	As required	Weight ≥1 kg below ‘green zone’	Manual	You’re below your green zone weight, congratulations! Why not set yourself some lower thresholds for your yellow and red zone to help keep the weight off? Please text me back with the new weights if you’d like to change them - if not, you don’t need to do anything. Thank you and well done.
Yellow	As required	As required	Participant declines to lose weight following regain		Thanks for telling us you’d prefer to avoid further weight gain, but don’t wish to lose weight at the moment. We are here to support and encourage you every step of the way. In this case, we reset your yellow zone weight to put you back into the green zone, then provide usual weight maintenance support. Are you happy for us to do this?
General					
Seasonal/date-based (e.g. 24 December)	All participants	On seasonal basis	Date	Automated	Many people decide to throw dietary caution to the wind on Christmas Day – don’t worry, we won’t try to dissuade you! It’s only one day of the year. However, if you fancy a delicious, indulgent Christmas Day that’s (surprisingly) under 2500 calories, then look no further: {link: web content}.

*If the input in question is not received within 24 hours (diary entry), a daily SMS reminder is sent until such input is received, or the participant opts out of receiving reminders. Minimum (possible) received SMS: 304; maximum (possible) received: 500 approx

*SMS* short message service

### *SMS* short message service

**Fig. 2:**
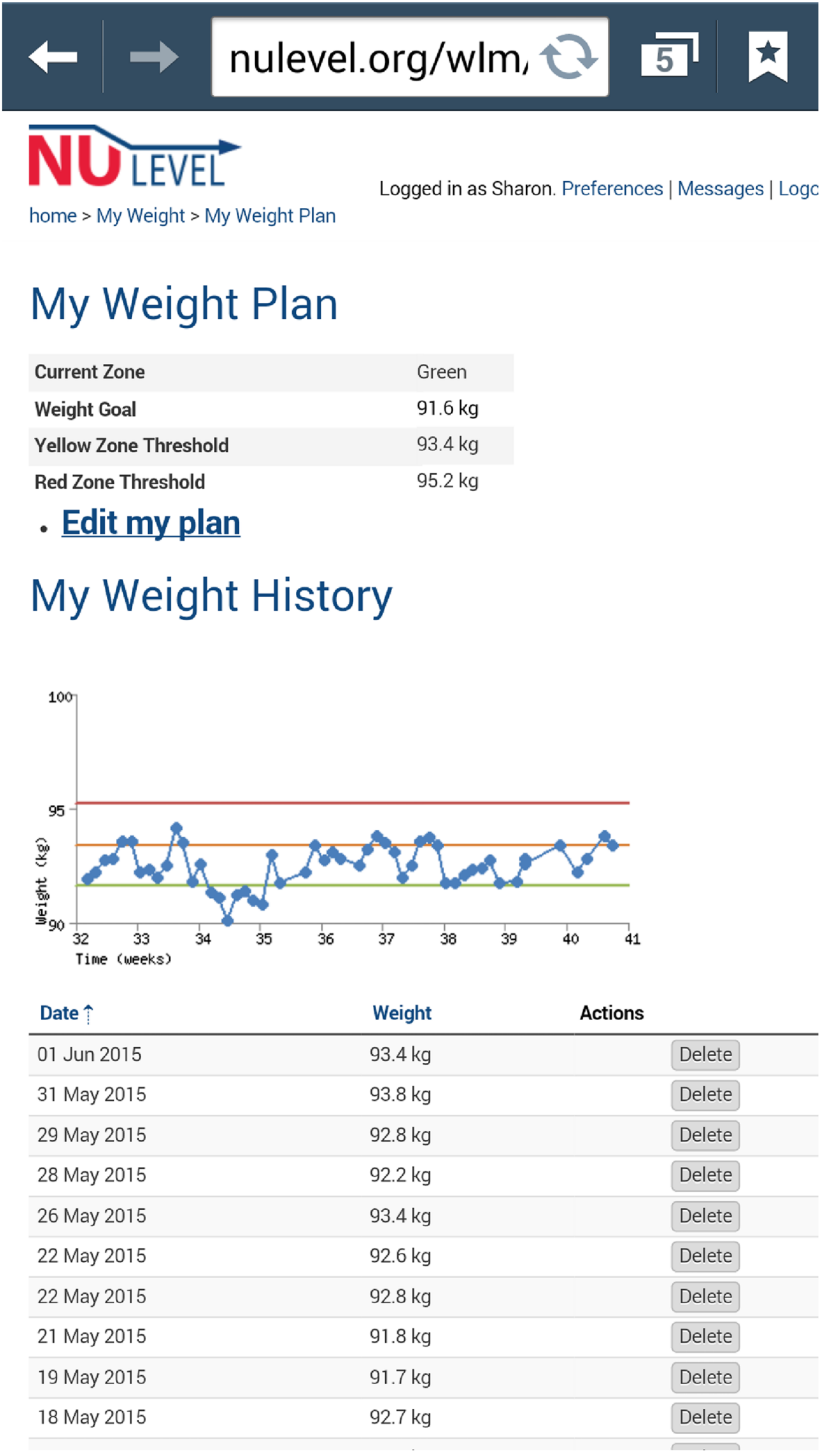
Screenshot of the NULevel mobile web interface

Participants are then introduced to the web-based intervention platform and shown how to enter information about their selected dietary behaviours and physical activity (step counts) against their goals in a weekly ‘diary entry’. Feedback on the diary entry prompts participants to reflect on their progress towards the personal dietary and activity goals, identified during the face-to-face WLM consultation; they are also prompted to rate their motivation and confidence to maintain their weight in the coming week. Figure 2 provides an example screenshot from the NULevel mobile web interface.

Participants are provided with a paper-based guide to self-monitoring their weight, physical activity and dietary progress, shown an example of a completed diary entry, and informed of their user name and password. They learn how the tailored SMS feedback allows them to evaluate their progress towards their outcome goal (weight) and behavioural goals (dietary and physical activity) in response to the information they enter each week. Finally, they learn how to edit, delete and create new dietary and physical activity goals for themselves.

### Daily weighing and weekly diary

Participants in the NULevel intervention are instructed to weigh themselves daily, ideally at a consistent time and under similar circumstances (e.g. in the morning after emptying their bladder, before eating or drinking and dressed in underclothes only). To establish this weighing habit, participants are prompted by text message to weigh themselves if no weight report is received from their scales for 24 hours in the first 2 weeks of the intervention, and for 72 hours or more thereafter. Daily measurements from each individual are evaluated for implausible day-to-day fluctuations, triggering a check on whether the measurement came from a non-participant, e.g. a family member. NULevel intervention participants are able to see the weights sent by their SIM-enabled scales displayed on a graph using the online platform.

Once a week, participants receive a prompt to complete their diary entry and an SMS link through which they can do so. Once they have submitted information regarding their step counts, achievement towards behavioural goals, confidence and whether they need additional contact with an intervention facilitator, they receive immediate feedback via on-screen messages regarding their dietary and physical activity goal progress. When the goals are repeatedly achieved, participants are prompted to set new ones. If the goals are repeatedly missed, participants are prompted to develop new plans to achieve the goals, or to set alternative, more achievable goals. This is supported within their web-based intervention platform.

### Automated and tailored remote feedback

Participants receive daily information and feedback from the study team. NULevel provides ‘light touch’ daily support in periods of successful WLM (i.e. while remaining in the green zone), which becomes more intensive during periods of weight gain (i.e. when moving into the yellow or red zones). Participants remaining in the green zone receive general tips and theory-based advice related to WLM as well as praise and reinforcement for keeping the weight off. If their weight enters the yellow or red zone, they receive tailored messages which support them to initiate a collaborative problem-solving process. This process is usually conducted via SMS between the research team member and the participant, but may include telephone calls if requested by the participant. Intervention content is based on six themes that were identified in a systematic review of behavioural theories [18] and in previous qualitative work [17] as relevant for behavioural change maintenance. These themes involve (a) sustainable motives for long-term WLM behaviours [27–30], (b) self-regulation and relapse prevention [25, 31–33], (c) management of personal resources [34], (d) habits and behavioural automaticity [30, 35, 36], (e) environmental and social influences [37] and (f) goal conflict, goal facilitation and priorities [38, 39].

If a participant’s weight falls into the red zone, they are sent, via mail, a personalised parcel of materials to support and motivate them in managing their regain, including a cook book, stress ball, personalised materials and leaflets containing advice and encouragement. Further details of NULevel intervention and the green, yellow and red zones are provided in Table 2.

### Control arm

For those allocated to the control arm, quarterly SMS messages are sent, containing links to evidence-based, weight management guidance. No further instructions regarding the use of the SIM-enabled scales are given. Participants can see their weight on the display of the scale, but no additional feedback on weight progress is provided. Participants are aware that the study team can access their weight data. There are no study-imposed constraints regarding the weight management behaviour of the control arm participants.

### Hypothesised mechanisms of impact

The NULevel intervention is hypothesised to reduce weight regain compared to controls through the following mechanisms:

1. The initial face-to-face WLM consultation is hypothesised to correct any existing misperceptions about a healthy and sustainable diet, establish clear behavioural goals, action plans and coping plans as well as to enhance self-efficacy for successful weight management and relapse prevention [40–42].
2. The provision of wirelessly connected scales, pedometers and brief weekly goal progress questionnaires via mobile internet is hypothesised to increase the rate of self-weighing, self-monitoring of behaviour, and the use of self-regulation strategies [43–46]. Prompting participants to develop routines of monitoring progress on weight, physical activity and eating behaviour goals is hypothesised to be a key change mechanism in weight management [15, 26]. Moreover, the regular self-monitoring allows individuals to focus their self-regulatory efforts on periods when WLM is more challenging, thereby conserving their psychological resources and avoiding intervention fatigue and ego depletion [34].
3. Theory-based text messages are hypothesised to support participants in focusing on motives which have been hypothesised to facilitate maintenance such as enjoyment of WLM behaviours [47], identity coherent with healthy lifestyle choices [46], self-determination [48], and satisfaction with weight outcomes [41]. Text messages also facilitate individual self-regulation [15, 43]and the development of healthy habits and routines [46]; they support the management of personal resources [34, 49], social and environmental challenges/opportunities [37], and goal conflicts and priorities [50].
4. Additional support in the yellow and red zone is hypothesised to help solving problems, managing temporary lapses and preventing relapses [32] as well as to provide social support [51].
5. Steps 1–4 are hypothesised to lead to healthier eating patterns [52], higher levels of physical activity and therefore reduced weight regain compared to controls. The maintenance of weight loss as well as the experience of control over one’s body weight are hypothesised to result in higher quality of life [53].

### Outcomes

The primary outcome is change in weight from baseline (i.e. randomisation) to 12 months. Secondary outcomes include weight-related anthropometric measures and self-reported measures related to the process and health economic evaluation of the study. A trained outcome assessor blinded to participant allocation status takes all anthropometric outcome measures and is present during the completion of questionnaire-based measures at the baseline and 12 months. An intermediate post-intervention assessment of questionnaire-based outcomes at 6 months is conducted via postal questionnaires. Table 3 summarises the study measures.

**Table 3:** Schedule of assessments for NULevel trial

Procedure	Visit 1: baseline (screening/randomisation)	Post-intervention assessment (6 months)	Visit 2 (12 months)
Informed consent	X		
Demographic details (and address)	X		
Blood pressure	X		X
Resting heart rate	X		X
Body weight	X		X
Height	X		
Body composition (hip/waist circumference; % body fat)	X		X
Physical activity (ActiGraph© GT3X+)	X		X
Economic assessments	X	X	X
Psychological assessments	X	X	X

Questionnaires were self-completed by participants. Assessors were trained to answer general questions

#### Anthropometric measures

Body weight, clothed without shoes, is measured by an outcome assessor, not involved in any other aspect of the study and blinded to the participants’ group allocation, to the nearest 0.1 kg using digital portable scales (SECA model 875) and height to the nearest 1 mm using a Leicester Height Measure stadiometer (both SECA UK Ltd: Birmingham, UK). BMI is calculated as body weight (kg) divided by the square of height (m). Waist circumference and hip circumference are recorded with an anthropometric tape measure, following established protocols [54]. Body fat percentage is measured using an Omron BF306 handheld body fat monitor, whilst resting heart rate and blood pressure are measured using an Omron HEM-7200 arm cuff automatic BP monitor (both Omron Healthcare UK Ltd: Milton Keynes, UK). Physical activity is assessed using an ActiGraph© GT3X+ (ActiGraph, LLC., Pensacola, FL, USA) accelerometer worn for at least 8 hours per day over 7 days. Height is measured once, at baseline; all other physical measurements are taken at baseline and 12-month follow-up.

#### Questionnaire-based measures

Participants complete a set of questionnaire-based measures at the baseline and 12-month follow-up visits in the presence of the trained outcome assessor. At 6 months, participants receive, self-complete, and return the questionnaire battery via post or online depending on their preference. This questionnaire includes measures of:

1. Health-related quality of life, assessed using the EQ-5D 3L [53] and healthcare costs and service usage, assessed using a structured questionnaire that was designed bespoke for this study from an existing item bank and a database of tools (www.dirum.ac.uk)
2. Satisfaction with weight outcomes, assessed by the Weight Outcomes Satisfaction Scale [41]
3. Self-efficacy for WLM, physical activity and healthy eating [42, 55]
4. Action planning and coping planning [40]
5. Eating attitudes and behaviours, assessed using the revised 18-item version of the Three-Factor Eating Questionnaire (TFEQ-R18) [52, 56]
6. Automaticity and identity, assessed by the Self-Report Behavioural Automaticity Index, also called the automaticity-specific Self-Report Habit Index subscale [46, 57]
7. Use of self-regulation strategies [43–45]
8. Ego depletion (a measure developed for this study)
9. Self-determination for weight control [48]
10. Social support, assessed using the ENRICHD Social Support Inventory [51]
11. Frequency and place of weighing (generic item)

Table 3 summarises the frequency and timing of assessments.

#### Process evaluation

A mixed methods process evaluation is being conducted to evaluate the implementation of the intervention and the mechanisms of potential impact. Furthermore, an examination of the effect of contextual factors on interventoin implementation and mechanisms is underway [58].

Quantitative indices by which process evaluation is undertaken include:

1. Timing and frequency of self-weighing in both trial arms (as automatically recorded by the SIM-enabled scales),
2. Usage data for intervention components, including completed phone calls, patient-initiated personal contact, frequency and duration of software module access, frequency of online eating and physical activity self-monitoring, number and nature of semi-automated intervention contacts) and
3. Secondary outcome variables (as assessed via the questionnaire-based measures) assessing theoretical intervention targets.

A qualitative process evaluation is also being conducted through theory-based semi-structured interviews [59, 60] with 40 randomly selected participants in their first 6 months in the trial following randomisation.

#### Fidelity assurance

Fidelity checks will be conducted to ensure consistency and adequacy of the face-to-face intervention sessions in delivering the specified session content and behaviour change strategies. A randomly chosen set of audio recordings of 20 initial advice/goal setting sessions and randomly chosen set of intervener-participant telephone contacts (summarised in written form using study-specific contact sheets) are being assessed. Interviews are transcribed verbatim. Data from the interviews and telephone contact sheets are entered into Nvivo and comparatively analysed against the intervention techniques [61] specified for the respective intervention component.

### Sample size calculation

The power analysis was informed by recent systematic reviews of RCTs of weight loss maintenance interventions [8, 9]. In order to detect a 2.5 kg between-groups mean difference in weight gain at 12 months, given a type 1 error rate of 5 % and assuming a standard deviation of weight gain of 6 kg with 90 % statistical power, two groups of 122 participants providing data on the primary outcome are required. Assuming a rate of 15 % loss to follow-up, a total sample of 288 randomised participants is needed. The parameters of this power calculation are derived from comparative behavioural WLM trials [13–15, 51]. The aim of the intervention is to flatten the typically observed weight regain curve.

### Randomisation and allocation concealment

Randomisation occurs in a 1:1 ratio (144 in each arm), and is stratified by sex and prior weight loss <10 % vs ≥10 %. A secure web-based randomisation system based on variable-length blocks, provided by Newcastle Clinical Trials Unit via Data Architects Ltd. (Newcastle upon Tyne, UK), is used to ensure concealment of allocation. The nature of the intervention is such that participants cannot be blinded, post-randomisation, as to their allocation status. Outcome assessors are blinded. When invited to attend follow-up assessments, participants are asked not to disclose their trial allocation to the outcome assessor. A record is kept to document all instances where participants reveal information about their trial allocation to the assessor.

### Data handling and record keeping

Data are being collected centrally and recorded automatically by a secure system devised specifically for this study. Data are being handled, digitised and stored in accordance with the Data Protection Act 1998. No participant identifiable data will leave the study site. Participants are identified within data collection systems by a unique identification number. The quality and retention of study data and data management is the responsibility of the Chief Investigator. All study data will be retained in accordance with the latest Directive on Good Clinical Practice (2005/28/EC) and local policy.

### Statistical methods

Specific analyses relate to each of the study objectives.

#### Effectiveness analyses

The main aim of the intervention is to help participants avoid regaining weight. The primary outcome is change in weight at 12 months post-randomisation relative to baseline. This will be analysed using analysis of covariance assuming a normal error structure. The dependent variable will be weight at 12 months post-randomisation. Baseline weight will be included as a covariate. Fixed effects will include two factors defining the stratification of the sample (sex of the participant and a binary indicator of whether the participant lost more than 10 % of their body weight) and a difference between the randomised trial arms (corresponding to an intention-to-treat analysis). Results will be in the form of a 95 % confidence interval for the mean difference in weight between participants randomised to the novel intervention and participants randomised to the control arm. If the standardised residuals are not normally distributed, an alternative confidence interval will be calculated using resampling (bootstrap) procedures. The impact of missing data will be assessed using a sensitivity analysis. Any data imputation will depend upon the level of missing data and its pattern. There will be no interim analysis. Secondary outcomes will be analysed using the same approach with an appropriate error structure adopted for each particular measure. Where appropriate, mediation analyses will be conducted to test if any observed effects of the NuLevel intervention are statistically accounted for by effects of psychological process measures.

#### Analysis of relationships between socioeconomic and demographic factors with WLM

The association between WLM and socioeconomic and demographic factors will be evaluated. The English Indices of Multiple Deprivation (IMD will be identified for each participant, matched to their seven-digit unit postcode. Other demographic variables will be collected in questionnaires at baseline. Relationships between age, sex, income band, education, IMD rank, employment status, car and home ownership, baseline BMI and pre-trial weight loss experience will be examined. We will extend the models used to estimate effectiveness with the inclusion of appropriate explanatory variables and interaction terms.

Data from exploratory analyses will be used to complement and inform the economic evaluation, thereby providing information to guide judgements as to how to reduce inequalities stemming from differences in effectiveness of WLM interventions and address necessary trade-offs between overall effectiveness and equity.

#### Health economics analyses

In order to estimate the cost-effectiveness of WLM in the RCT, a within-trial economic analysis will estimate the incremental cost per unit difference in weight regain. Costs will be based upon intervention costs (actual) and costs of any additional services used or incurred by participants. With respect to the intervention costs we aim to develop this intervention so that it could be delivered to the >93 % of the population that currently owns a mobile phone, for under £100 (+ costs of personal contact). Analyses will draw on data from the Healthcare Usage Questionnaire and quality-adjusted life years (QALYs) will be estimated using the EQ-5D 3L. Extrapolation beyond the trial will be based upon a state transition model drawing on previous work of the co-applicants. The results will be presented as an incremental cost per QALY gained. Costs, health state utilities and the probabilities of future events required to populate the model will be based upon our prior work and structured reviews of the literature. Both probabilistic (to explore the impact of statistical imprecision) and deterministic models (to explore other uncertainties and scenarios – e.g. the impact of a less intensive intervention) will be explored. The perspective of the analysis is the National Health Service (NHS), other care providers and the participant.

### Compliance and withdrawal

Compliance with outcome assessment is evaluated by the outcome assessor and by the completion rates of the study questionnaires. Compliance with the intervention is assessed via the frequency of self-weighing, attendance at the intervention session and by usage statistics for the intervention platform, as accessed by participants as part of the digital component of the intervention. Standard operating procedures for dealing with non-responders and with participant withdrawal are followed (Appendix A).

### Internal pilot study

An internal pilot study to evaluate the acceptability and feasibility of the trial procedures (e.g. recruitment, measurement, allocation and retention procedures) was completed in July 2014 before proceeding with the full trial. Progression to a full trial required that, in the first 3 months of the trial, the team (1) recruited, consented and randomised ≥50 participants, (2) established evidence, through participant feedback, of the acceptability of trial procedures, and (3) sustained less than 20 % participant loss to follow-up in data collection. All three targets were met, which allowed continuation to the full trial without changes to the protocol. Therefore, the data obtained during the internal pilot period will form part of the trial dataset.

## Discussion

This is the first randomised trial to test the effectiveness of a WLM intervention utilising ubiquitous technology to tailor an evidence-informed intervention to obese adults who have lost a clinically significant amount of their body weight. The study includes an economic cost-effectiveness evaluation. This trial will provide policy makers and commissioners with the evidence to decide whether to adopt this method to support obese adults who have lost weight. Cost-effective WLM interventions are a vital complement to existing services targeting weight loss, including commercial weight management programmes. They also potentially benefit healthcare services that directly and indirectly target obesity, and cope with its consequences.

The current study addresses research needs identified by a recent systematic review [9, 62]:

1. All but three [15, 22, 23] RCTs identified in this review included an initial period of facilitated weight loss, mostly through very low-energy diets. While this procedure is practical for research, it might limit generalisability and does not easily translate to a public health prevention approach.
2. Most effective WLM interventions to date have been highly resource intensive and hence not scalable, i.e. practical for widespread implementation.
3. Previous attempts to use technology to make interventions flexible, efficient and scalable have not used contemporary digital technologies. Interventions using the internet for intervention delivery were not effective [15, 63–65].

In the NULevel trial, the first research gap is addressed by including participants drawn from a wide range of settings, to more closely reflect weight management practices of the population. This includes participants engaged with commercial and community-based weight loss programmes as well as those who lost weight independently of professional support . The second and third main research gaps are interrelated because effective technology use could help to make interventions both more accessible and affordable. Rather than delivering interventions *if and when* participants use their computer to browse the internet, mobile phone platforms provide more promising modes of delivery because they allow immediate access to information in any situation and context. Feature phones (i.e. standard mobile phones with access to the internet) and smart phones are ubiquitous in both developed and developing economies, and provide a robust technology platform for cost-effective delivery and rollout of interventions. In the UK, 93 % of the adult population owns a mobile phone that can be used to access the internet and coverage is good even among the most deprived households (87 %) and in the 75+ age group (58 %). These figures are rising and compare very favourably with access rates to home computers with internet connectivity (71 % of UK households; 54 % in the most deprived households; 25 % for 65+ age group) [66]. There is good evidence for the acceptability and effectiveness of mobile phone-delivered interventions from weight loss studies [67–69].

The NULevel trial will inform how best to encourage and enable WLM in obese adults who have successfully lost weight. It will show if a theory- and evidence-informed intervention based on interactive digital technology is cost-effective in supporting WLM. Further, it will indicate whether the resulting NULevel intervention platform is an efficient means of utilising limited resources (such as staff time) to optimise outcomes in populations, and what level of additional personal support is needed to obtain optimal outcomes per investment.

Obesity remains a growing and intolerable burden on services and society, and thus finding cost-effective means to help sustain effects of weight loss interventions is vital for international public health.

## Trial status

The trial described in this protocol is now at the recruitment, post-pilot stage.

## Abbreviations

BMI: body mass index
IMD: Index of Multiple Deprivation
NHS: National Health Service
QALY: quality-adjusted life year
RCT: randomised controlled trial
SMS: short message service
UK: United Kingdom
US: United States
WLM: weight loss maintenance

## Footnotes

1 The technology for the scales has been released as WeighAX, part of the Open Movement open-source project (http://openmovement.co.uk).
